# Correction: Real environment obstacle circular edge expansion design robot path planning based on ant colony algorithm

**DOI:** 10.1371/journal.pone.0357379

**Published:** 2026-08-31

**Authors:** Feng Li, Maoya Yang, Seong-Nam Jo, Young-Chul Kim, Ziang Lyu

The images for Figs 21 and 22 are incorrectly switched. The image that appears as Fig 21 should be Fig 22, and the image that appears as Fig 22 should be Fig 21. The figure captions appear in the correct order. The authors have provided a corrected version of the figures here.

**Fig 21:**
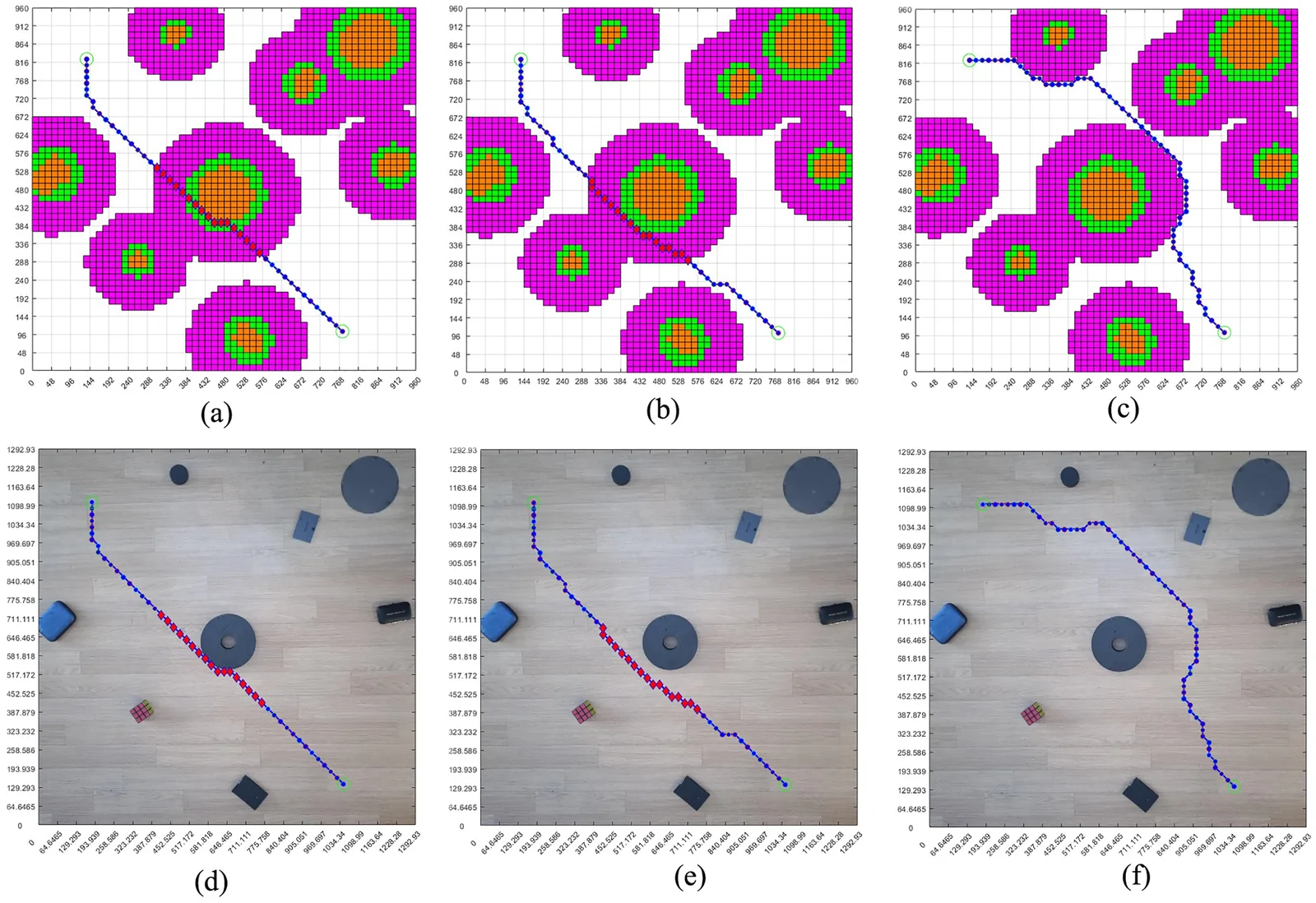
Real environment planning path security. **(a)** Real environment grid map planning path security. **(b)** Circular design of real environment obstacles grid map planning path security. **(c)** Real environment obstacle circular edge expansion design grid map planning path security.

**Fig 22:**
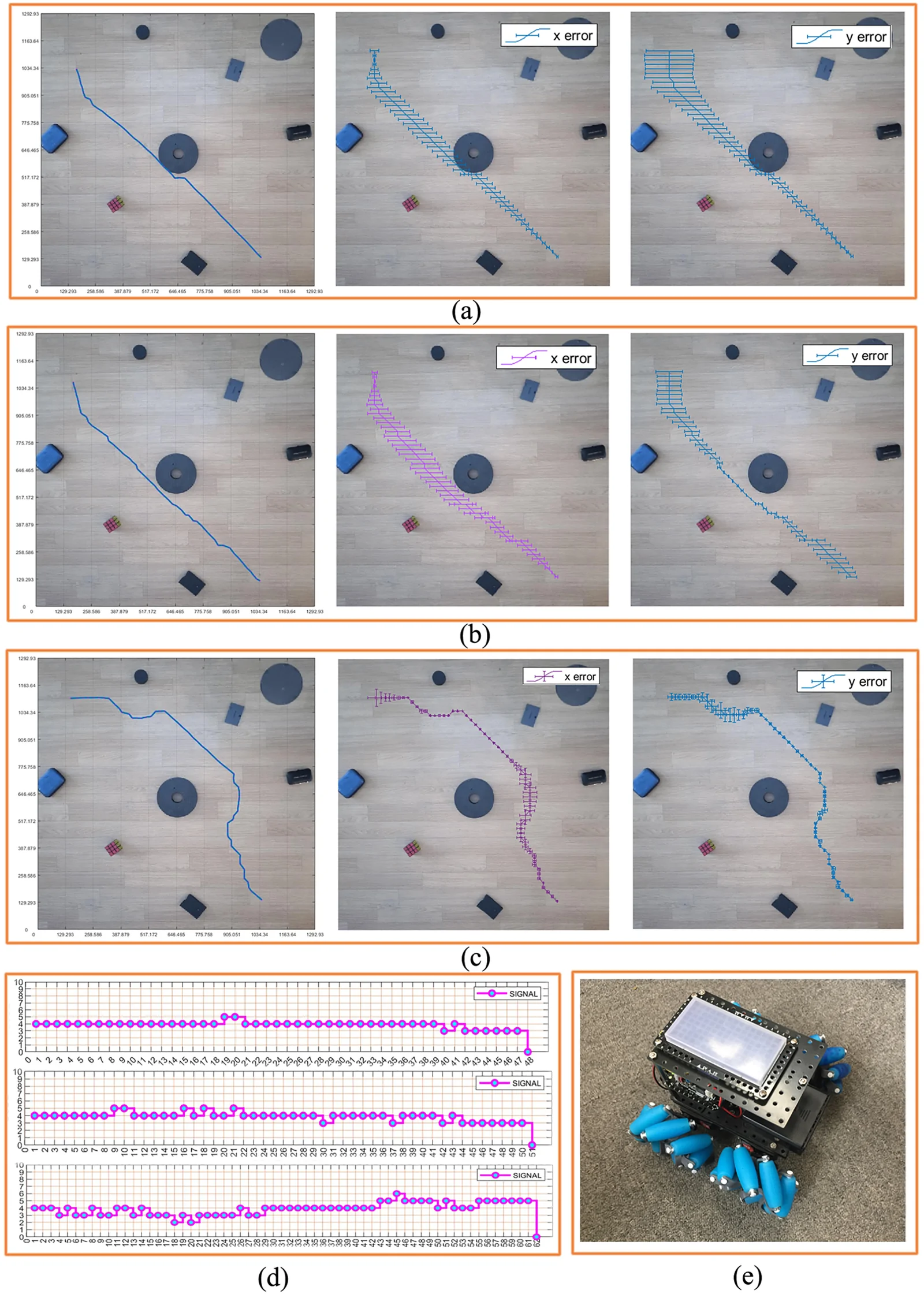
Real environment robot motion control experiments. **(a)** Real environment grid map planning path motion control experiment actual trajectory, x direction error, y direction error. **(b)** Experiment actual trajectory, x direction error, y direction error of the real environment obstacle circular design grid map planning path motion control experiment. **(c)** Experiment actual trajectory, x direction error, y direction error of the real environment obstacle circular edge expansion design grid map planning path motion control experiment. **(d)** Coding graphs under three types of maps. **(e)** Experiment robot.
