# Supplementary material for: Real environment obstacle circular edge expansion design robot path planning based on ant colony algorithm
Source: PLoS One. 2026 May 7;21(5):e0348580. doi: 10.1371/journal.pone.0348580 (PMC13152169; doi:10.1371/journal.pone.0348580)
Supplement: Attachment — Submitted filename: Response to Reviewers.doc [file pone.0348580.s004.doc]

**Dear Editor,**

Thank you for your review. In response to the suggestions provided, we have added relevant diagrams and tables. We have included the results of the motion control experiment, path planning experiment, and path planning of other algorithms, as well as the results of runtime decomposition. Since the motion control experiment was only intended to verify the effectiveness of the path planning, we did not conduct more research on the robot's movement. Additionally, the initial position and target position were set to make the planning area more extensive, and this does not have a strict requirement for the repeatability of the path planning. The specific supplementary and improved diagrams are as follows.

In addition, we have also improved the other figures in the text. We have also refined the coordinate systems of the figures in the text and unified the coordinates. Please refer to the question for details.

**Reviewer #1:**
**1- The real-world evaluation appears to rely on a single tabletop layout (7 obstacles + 1 reference) captured by an overhead phone, with one chosen start–goal pair (codes 66 → 3538). This is not sufficient to establish robustness. Please repeat the full pipeline on multiple real environments (vary obstacle densities/shapes, start–goal pairs, and robot sizes) and report aggregate statistics (success rate, min-clearance, path length, tracking error, collisions). The current description shows one map being standardized (Fig. 13–14/16–18) and then used for the three comparative cases only.**

Dear reviewer, thank you for your review. To address your comments, we have added the path planning and motion control experiments (as shown in Fig 25 and Table 7). Additionally, we have also added the path planning in different non-standard environments and demonstrated the path planning results (as shown in Fig 27). Since this paper focuses on the path planning in non-standard real environments, the robot's path tracking mainly studies the control accuracy of the robot system, and is not the end point of our research. The path planning in different environments can precisely illustrate the effectiveness of the methods proposed in this paper. Thank you for your guidance. In our subsequent research, we will conduct more in-depth studies on the robot itself.

**2- Fig. 21/22 present x/y error and pose error “box diagrams,” but the manuscript reports only qualitative impressions (e.g., “median generally lower than 5°,” “y-error about 8”). Please tabulate the exact medians, IQRs, and RMS/MAE for x, y, and pose, and clarify how “abnormal points” (outliers) were identified/removed. Without explicit numbers and outlier policy, the comparison across the three maps is hard to interpret.**

Thank you for your suggestion. This paper mainly focuses on path planning using non-standard real-world environment maps. The table is provided merely to demonstrate that the path planning conducted using the methods presented in the paper can be applied to the motion control of robots. We hope to prove the feasibility and effectiveness of the proposed methods through experiments. The research on the accuracy of robot motion control is mainly about the study of robots, rather than the main focus of this paper. Therefore, we did not elaborate much. Please understand. Also, thank you for your reminder. We will conduct further research on the robot system in the future to effectively improve the motion control accuracy of the robots.

**3- In the virtual-environment test, the authors note “17 dangerous points” along the planned pose before expansion; that’s useful, but please (i) state the exact δ used to classify danger per Eq. (19), (ii) report the count/min-clearance for all three maps in both virtual and real experiments, and (iii) provide distributions of point-to-obstacle distances along the final paths. A small summary table would make the improvement unambiguous.**

Dear reviewer, thank you for your review. To further improve the article, we have added relevant descriptions and provided Figure 21. Please review them. Through these improvements, the research significance of this article has been effectively enhanced. Thank you very much for your guidance.

**4- You report “from original map to Fig. 18 used 23.075115 s.” Please decompose this into per-stage times (binarization, obstacle detection, circularization, edge expansion, gridding, ACO planning), list hardware/OS and software versions, and provide** **averages ± SD over ≥10 runs. Also report ACO convergence iterations/time for each of the three maps.**

Thank you for your review. Based on your suggestions, we have added Tables 5 and 6. Please review them. Through these improvements, the research significance of this article has been effectively enhanced. Thank you very much for your guidance.

**5- All comparisons hold the planner fixed (ACO) and vary map preprocessing (raw vs circular vs circular+expansion). For completeness, please add at least one classical baseline (e.g., A*, D*, RRT/RRT*) on the same three maps and report the same metrics (path length, min-clearance, collisions, planning time). This will show whether the claimed safety/robustness benefits persist beyond a single metaheuristic. (This request is motivated by the paper’s exclusive reliance on ACO in the current Results.**

Dear reviewer, thank you very much for your guidance. Since path planning using non-standard real-world environment maps is a very large topic, in this paper, only ACO was used for path planning, which demonstrates the feasibility of the proposed method. To further improve the content of this paper, the research team also used maps to conduct path planning with methods such as A*, RRT, and PRM. Since the D* algorithm was not used by the research team, it was not presented. Please refer to Figure 26 and Table 8 for details. These figures and tables illustrate the feasibility and effectiveness of the methods. Due to the different operation methods of various algorithms and the related parameters of other algorithms involving the publication and contribution of other members, it is not convenient to provide them. Please understand.

**6-** **The “time coding graph” approach implies mostly open-loop pose execution; please detail the controller (kinematics of the four omni wheels, sampling period, speed limits, any odometry/IMU/vision feedback), the error computation method (in what frame? units on axes?), and how the time codes were generated from poses. These details are essential to interpret the tracking error plots in Fig. 21/22 and to reproduce the experiments. Also state whether wheel slip/friction was characterized.**

Dear reviewer, the "time coding graph" is an encoded graph generated from the pose graph to control the movement of the robot. The robot has 8 movement directions in each grid, and each direction is assigned a code, as shown in Figure 22. The time coding graph enables the robot to maintain a signal for a certain duration for each encoded signal. This duration is equal to the length of the grid divided by the robot's movement speed. To better illustrate the principle of the encoding, we have provided a pose encoding graph, as shown in Figure 23d.

The position deviation of the robot's movement is, on the one hand, caused by the accuracy of the robot's movement itself, and on the other hand, caused by the friction and slipping of the robot on the ground when it moves. The robot motion control experiments in the text are designed to verify the feasibility and effectiveness of the proposed planning path using the non-standard environmental map in the real environment. In fact, the figures 21-27 presented in the text have already demonstrated the feasibility and effectiveness of the planning path. We have verified the planned path and pose using the robot for experiments, which not only enriches the research content of this paper but also re-verifies the feasibility and effectiveness of the proposed path and pose planning in the real environment using non-standard environmental maps.

The items you mentioned (kinematics of the four omni wheels, sampling period, speed limits, any odometry/IMU/vision feedback) are mainly about the research of the robot. We have conducted relatively few studies on the robot itself, and our research mainly focuses on its applications. Thank you for your reminder. We will also conduct an in-depth study on the robot itself in the future.

**7- BLC is computed from a 200 mm reference (REDMI 9A overhead capture), yielding BLC = 0.7425. Please report** **calibration uncertainty (lens distortion, perspective, height variation), how BLC propagates to path-length and clearance errors, and whether the min-clearance** **threshold δ includes a calibration safety margin. Without this, the mapping from pixels to millimeters in the Results remains uncertain.**

Dear reviewer, thank you for your review. The issues you raised have been addressed and explained in the text as follows. It is precisely because of the calibration uncertainty that we included the safety distance when calculating the threshold δ. In fact, we can directly observe the rationality of the planned path and its effectiveness from Figures 20, 21, 25, and 27. The path planning effect can be clearly seen from the figures, and it is very good. Thank you for your reminder. We will also conduct relevant research on this issue in the future to improve the path planning effect. Since path planning in real environments is a very large topic involving many research directions, thank you for your guidance. We will continue to improve and optimize in the future.

Reviewer #2:
Thanks for sharing—there’s a very useful idea here. That said, in its current form it needs a major revision.

**1.The author define** **J = floor(B/255) and then explain “<255→0, =255→1” (L158–L162). That basically treats only pure white as free space—too brittle. Use a real threshold (Otsu or EDT→threshold), report T and robustness.**

Dear reviewer, thank you very much for your valuable comments. Utilizing J = floor(B/255) to binarize the matrix is a common method for converting a map into binary form. Each researcher can set it according to their own requirements. You can also use the ROUND function, but usually this method is employed. We can further expand the boundary of the obstacles by adding a safety distance and other methods to ensure the safety of the planned path.

Dividing the obstacle boundaries using a computer is relatively accurate. In fact, if the computer cannot distinguish the boundaries, in real scenarios, it is even more difficult for the human eye to distinguish the boundaries of the obstacles. Since the method proposed in this paper is for path planning in non-standard real environments, and the topic of path planning in non-standard real environments is very large, there are many subsequent studies that need to be conducted, including the method you proposed for distinguishing the boundaries of the obstacles. Thank you very much for your guidance. Your guidance has also provided us with a new research direction, and our research group will continue to explore in this direction in the future.

**2.The author first set origin at top-left, +x right, +y down (L147–L149, L172–L174), but later you switch to left→right & bottom→top (L191). Pick one convention and rewrite the encode/decode formulas accordingly.**

Dear reviewer, thank you for your review. We didn't notice this issue in the early stage. Therefore, we have improved all the figures in the article, agreed on the setting principles, and the improvements can be seen in the figures within the article.

**3.The author state the rule Lij ≤ δ (unsafe) and Lij > δ (safe) (L269 & L273), and you expand obstacles with Rsi = (LCol + LCow)/2 + Ls (L305–L307), but there’s no calibration of δ / Ls (units, how chosen, sensitivity). Define δ = robot radius + sensing/pose error margin, implement via configuration-space dilation / EDT, and show a quick sensitivity check.**

Dear reviewer, thank you for your review. The setting of δ has been described in the text as follows:

Because δ has already been incorporated into the map during its generation, that is, it has expanded the circle, as shown in the figure:

We deformed the obstacles in this way to ensure that the safety distance was included during the design process. Therefore, this δ is not for checking purposes, but is a requirement that needs to be met when designing the obstacles. We demonstrated the planned path in the real environment and verified the effectiveness of the planned path again, as shown in the following figure.

**4.The author list ACO params only (K=100, M=100, α=1, β=30, ρ=0.2, Q=100) (≈L343–L351), claim 23.075115 s end-to-end (L427), but give no HW/SW context or manual/auto breakdown, no baselines (A*/D* Lite/RRT*), and later show a “Safety degree change graph” (L533) without a mathematical definition. Report** **path length, minimum clearance, runtime (mean±SD/CI), add baselines, define “safety” formally, and back the claim with stats.**

Dear reviewer, thank you for your review. In the article, it has been pointed out in the forefront that currently, the main methods are to obtain real environmental data through self-modeling or through LiDAR, and then model based on the real environmental data. Therefore, there are difficulties in path planning. If only using a non-standard real environmental map for path planning, the difficulty will be greatly reduced and the intelligence level will be greatly improved. This is the significance of this article, and the safety change graph of this article is also one of the research methods explained in this article. Since there is no reference for comparison, there are no baselines as you mentioned. To address your question, the research group has made the following supplements in the article:

(1) By conducting path planning in other algorithms (A*/PRM/RRT/GA/Dijkstra/APF), the feasibility and effectiveness of the path planning using non-standard real environmental maps proposed in this article are illustrated, as shown in the following figure.

(2) The concept of safety level was explained, and relevant diagrams were also provided.

(3) According to the explanation, we have presented the statistical results in Figure 24 below, as shown in the figure.

Thank you very much for your attention. As the research topic is in a relatively new field, there are insufficient relevant references. However, the proposal of the method in this paper has extremely significant meaning and serves as a valuable reference for the research on global path planning methods in real environments.

Reviewer #3:
**The contribution here is very incremental,** **obstacle expansion and ant colony optimization are not really new ideas.** **The experiments are small scale and very controlled, no replication or solid statistical robustness is shown.** **Also no comparisons with standard baselines (A*, D*, RRT etc) so it’s hard to judge if this method is actually effective. T****he claim of “100% safety” is overstated, not supported with strong evidence. The language quality is weak, lot of grammatical errors, awkward phrasing, even typos.** **Figures feel repetitive and captions don’t explain clearly.** **Data availability is also not enough for reproducibility, no raw trajectories, code or scripts provided. Overall, the presentation falls short of journal standards.**

Dear reviewer, regarding your review comments, I provide a detailed explanation below. Thank you very much.

**（1）“The contribution here is very incremental, obstacle expansion and ant colony optimization are not really new ideas. ”**

Dear reviewer, current research mainly takes place in virtual environments or obtains real environmental information through lidar and then builds an environmental map. However, there are few studies on conducting global path planning research using non-standard environmental maps of the real environment. This is the core contribution of this paper. And this has a very significant importance for the research on robot path planning, greatly enhancing the intelligence level of robots.

**（2）The experiments are small scale and very controlled, no replication or solid statistical robustness is shown.**

Dear reviewer, our research team conducted numerous experiments, but only a small portion of them are presented in the paper. Since the topic of real-world environments versus standard environment maps is a very broad one, this paper theoretically explains it through virtual non-standard environment maps and verifies it through real non-standard environment maps. In response to this comment, we have included Figures 25, 26, and 27 in this paper for partial presentation. These can demonstrate the effectiveness of the method and we hope for your approval.

**（3）Also no comparisons with standard baselines (****A*, D*, RRT etc) so it’s hard to judge if this method is actually effective.**

Dear reviewer, as this paper introduces a method for path planning in a real environment using non-standard real environment maps, this method is mainly aimed at converting the non-standard environment maps of the real environment into grid maps that can be used for robot path planning. The use of the ant colony algorithm is just one application of this method. Therefore, no other methods' applications were added. In response to this comment, the research team has added the path planning effects of multiple algorithms. The specific situation is shown in Figure 26. Thank you for your reminder.

**（4）The claim of** **“100% safe****ty” is overstated, not supported with strong evidence.**

Dear reviewer, the article states that this method can theoretically ensure "100% safety" of the planned path. This is because adding a safety distance outside the robot is precisely to ensure "100% safety" of the planned path. If the principle does not guarantee "100% safety" of the planned path, then the research in this article would be meaningless. This is one of the purposes of this article and also the significance of the research. In response to your comments, additional figures have been added in the article to explain this. Thank you for your suggestions.

**（5）The language quality is weak, lot of grammatical errors, awkward phrasing, even typos.**

Thank you for your review. To improve the quality of the sentences, the research group has once again had them reviewed by a foreign language professional teacher, and has also used the "Grammarly" software to check the sentences and words. The modified parts of the sentences in the text have been marked in the paper. Please review it. Thank you for your guidance.

**（6）Figures feel repetitive and captions don’t explain clearly.**

Dear reviewer, thank you for your review. Regarding this point of opinion, the research team feels that you might be referring to the similarity between path planning and pose planning diagrams. Here, it should be pointed out that since path planning shows the movement path of the robot, and pose planning shows the various positions and poses that the robot needs to pass through during its movement, the combination of the two can better demonstrate the effect of path planning in non-standard real environment maps.

**（7）Data availability is also not enough for reproducibility, no raw trajectories, code or scripts provided.**

Dear reviewer, thank you for your review. We have provided the running parameters in the attachment for your review. The theoretical and practical explanations in the text illustrate the repeatability of this method. Additionally, we have also supplemented the planning results under various scenarios and various algorithms, once again demonstrating the repeatability of this method.

Since path planning in non-standard real environments is a very complex topic, the research team has invested a great deal of financial resources, materials, and manpower in this study. There is still a lot of research to be conducted in the future. Therefore, the code cannot be provided now. Thank you for your understanding.
